# Breast Cancer Metastasis in a Renal Carcinoma Pulmonary Metastasis: A Rare Example of Tumor-to-Tumor Metastasis

**DOI:** 10.1155/2021/3054232

**Published:** 2021-06-23

**Authors:** Áurea Lima, Isa Peixoto, Susana Sarandão, Daniel Melo, Ângelo Rodrigues, Helena Pereira

**Affiliations:** ^1^Medical Oncology Service, Centro Hospitalar de Entre o Douro e Vouga, EPE, Unit of Santa Maria da Feira, R. Dr. Cândido Pinho 5, 4520-211 Santa Maria da Feira, Portugal; ^2^Molecular Oncology and Viral Pathology Group, Research Center of Portuguese Institute of Oncology of Porto, Portuguese Institute of Oncology of Porto Francisco Gentil, EPE, Rua Dr. António Bernardino de Almeida, 4200-072 Porto, Portugal; ^3^CESPU, Institute for Research and Advanced Training in Health Sciences and Technologies, Cancer Research Group, R. Central da Gandra, 1317 Gandra, Portugal; ^4^Medical Oncology Service, Centro Hospitalar Universitário do Porto, EPE, Largo Prof. Abel Salazar 4099-001 Porto, Portugal; ^5^External Radiotherapy Service, Portuguese Institute of Oncology of Porto Francisco Gentil, EPE, Rua Dr. António Bernardino de Almeida, 4200-072 Porto, Portugal; ^6^Pathologic Anatomy Service, Centro Hospitalar Universitário de São João, Alameda Prof. Hernâni Monteiro 4200-319 Porto, Portugal; ^7^Pathologic Anatomy Service, Portuguese Institute of Oncology of Porto Francisco Gentil, EPE, Rua Dr. António Bernardino de Almeida, 4200-072 Porto, Portugal

## Abstract

The tumor-to-tumor metastasis phenomenon remains fairly uncommon, with fewer than 100 cases described to present time. Virtually any tumor can be a donor or a recipient neoplasm. Nevertheless, renal carcinomas have been implicated as the most common malignant tumors to harbor metastasis, while lung and breast tumors are the most frequent donors. This article reports an extremely rare case of a breast cancer metastasis in a lung metastasis of clear cell type renal cell carcinoma that met all Campbell and coworkers' tumor-to-tumor metastasis criteria. Additionally, we present the literature case reports of breast cancer metastasis in renal cell carcinomas and try to discuss the mechanisms underlying its occurrence. Since this phenomenon identification will impact the therapeutic strategy and it is not easily detected by image, the anatomopathological study of any and all suspicious lesions is of crucial importance. To the best of our knowledge, this is the first report of a metastasis inside a metastasis.

## 1. Introduction

Tumor-to-tumor metastasis (TTM) is a term describing the presence of two histologically distinct tumors at one location, each with different morphologic and immunophenotypic features [1]. It is an extremely rare phenomenon in patients with multiple synchronous or metachronous primary malignancies. The most common metastatic donor neoplasms are lung and breast cancers, while meningioma is the most common recipient of metastasis among benign tumors and renal cell carcinoma among malignant tumors [2]. Although the lung is one of the organs most vulnerable to metastases, a lung carcinoma is one of the rarest recipients of TTM [3]. Furthermore, the phenomenon of a metastasis in another metastasis is still a rarer event.

Herein, we report a TTM case of a breast cancer metastasis in a pulmonary metastasis of clear cell type renal cell carcinoma (ccRCC).

This work has been approved by CHEDV's Ethics Committee, according to the Helsinki Declaration of the World Medical Association; also, the patient gave written informed consent to have her case used for this paper.

## 2. Results

### 2.1. Patient History and Presentation

**Figure 1:**
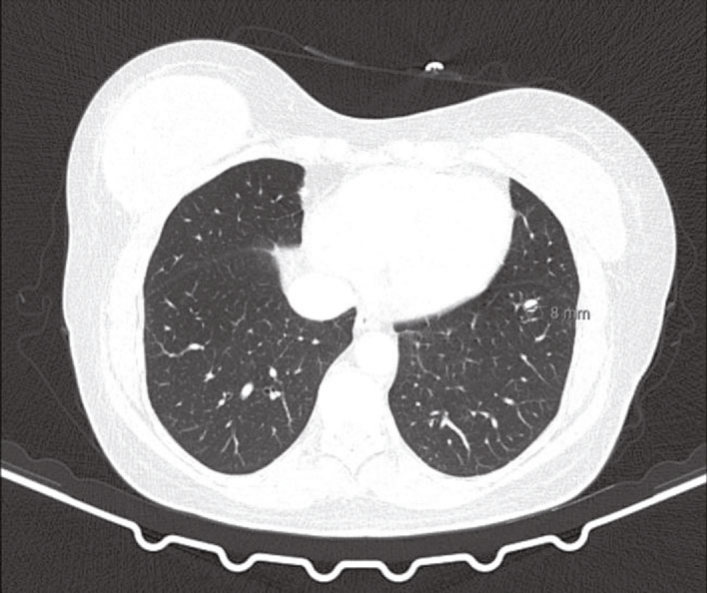
Transverse section of a thorax CT scan presenting an 8 mm pulmonary nodule.

We present a 57-year-old woman with a past medical history of stage IV ccRCC (pT3aR0cN0M1) diagnosed in August 2019. During diagnosis and staging evaluation, she was observed to have a 12 mm suspicious lesion on the left kidney and a 20 mm nonsuspicious solid nodule in the right adrenal; the multidisciplinary team (MDT) decided left radical nephrectomy, performed in September 2019, plus clinical and imagological surveillance of the right adrenal nodule. Follow-up exams revealed an increment of 2 mm of the right adrenal nodule without new lesions; MDT decided histological evaluation of the lesion, which revealed a ccRCC metastasis. Since this was a unique metastasis, a right adrenalectomy was performed in February 2020. In March 2020, the patient referred to having a palpable nodule in the left breast. The auxiliary diagnostic tests carried out revealed a stage III invasive ductal carcinoma (IDC) ER 100%, PgR negative, HER2 positive (FISH), and Ki67 > 30%, initially managed with neoadjuvant chemotherapy (CHT) with sequential anthracycline/taxane-based regime combined with dual anti-HER2 blockade (trastuzumab/pertuzumab), followed by surgery (cT3N+/ypT2N1(3/15)cM0). In November 2020, the follow-up CT scan (Figure 1) showed bilateral pulmonary nodules.

### 2.2. Diagnostic Workup

**Figure 2:**
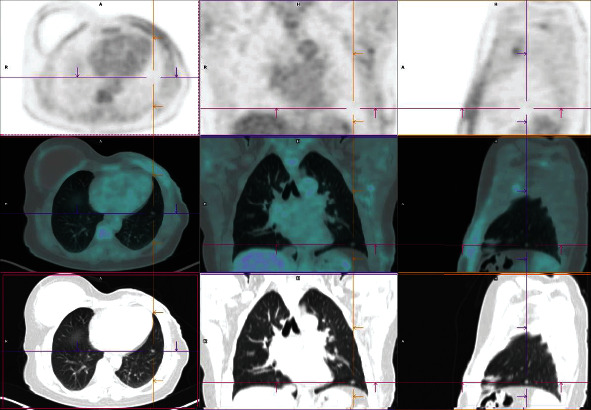
PET/CT images showing an increased metabolic activity in the pulmonary suspected lesion.

The patient was asymptomatic, with a Karnofsky performance status (KPS) score of 100. No positive findings were encountered in the anamnesis on physical examination and/or other follow-up exams. PET/CT showed abnormal 18F-FDG uptake in the referred suspected pulmonary nodules, without other hypermetabolic changes that may suggest malignant involvement (Figure 2).

**Figure 3:**
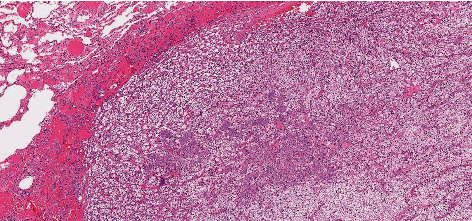
Microscopic examination of the basal nodule biopsy specimen (H&E, 40x). Pulmonary parenchyma (upper left corner) with involvement of epithelial cells with the clear cytoplasm and well-defined cell membrane, interspersed within a highly vascularized stroma, compatible with ccRCC. Inside this, a distinct population cells, arranged in nests, with a more eosinophilic cytoplasm is identified, corresponding to the breast carcinoma.

**Figure 4:**
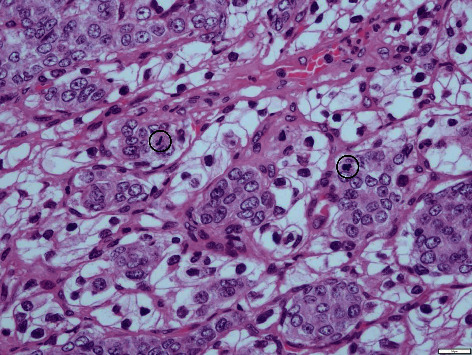
Microscopic examination of the basal nodule biopsy specimen with a high-power field showing the two neoplastic components (H&E, 400x). Note the two mitotic figures on the breast cancer component.

**Figure 5:**
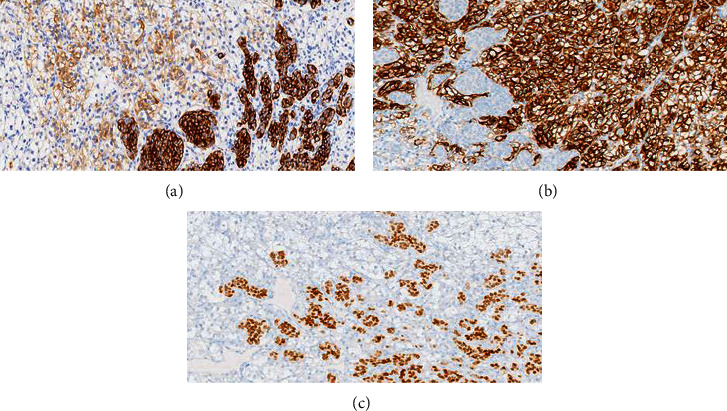
Immunohistochemical stains. (a) (Cam5.2, 200x) Both neoplasms are positive for cytokeratins, confirming an epithelial origin of the two neoplastic populations. (b) (CD10, 200x) Strong and diffuse positivity for CD10, characteristic of ccRCC. Highlight the antibody's negativity in the breast carcinoma cell population. (c) (ER, 200x) Strong and nuclear positivity for the antibody directed to ER in breast carcinoma cells. Highlight the antibody's negativity in the ccRCC component.

Given the impossibility of performing needle biopsy, the patient was proposed and accepted for surgery. Three pulmonary nodules, located in the left lung (basal, cisural, and upper lobe), were excised. Microscopic examination was performed to all nodules. Cisural and upper lobe nodules corresponded to ccRCC metastasis without additional findings. Microscopic examination (Figures 3 and 4) and immunohistochemical study (Figure 5) of the basal nodule biopsy specimen revealed two distinct neoplasms. The following immunophenotype was observed: CD10+, vimentin+ (heterogenous), MelanA-, inhibin-, CK7-, and ER-, and inside this, another distinct phenotype was observed: CD10-, vimentin-, MelanA-, inhibin-, CK7+ (few cells), ER+, PgR-, GCDFP15-, Cam5.2+, EMA+ (few cells), and CD56-. Therefore, the presented results raised the hypothesis of being a breast cancer metastasis in a lung metastasis of ccRCC.

### 2.3. Medical Management and Treatment

**Figure 6:**
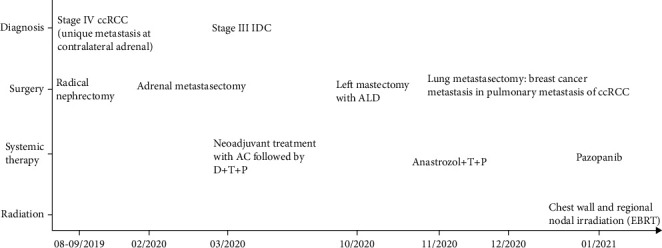
Overview of the patient clinical history and management.

Concerning the breast cancer, breast MDT proposed the patient, in November 2020, for adjuvant radiotherapy plus hormone therapy with anastrozole and dual anti-HER2 therapy with trastuzumab/pertuzumab (the anatomopathological confirmation of the breast cancer metastasis in the lung metastasis of the ccRCC occurred later in December). As for the ccRCC, urologic MDT proposed the patient, in January 2021, to pazopanib. An overview of patient clinical history and management is given in Figure 6.

## 3. Discussion

Metastasis from one neoplasm (the donor) to another neoplasm (the recipient) has been described in the literature for many years since Fried published the first documented case of bronchogenic carcinoma metastatic to a meningioma in 1930 [4, 5]. In 1968, Campbell and coworkers proposed the following basic criteria for the diagnosis of TTM: (i) diagnosis of more than one primary tumor; (ii) extravascular metastasis existence; (iii) confirmation that it has not resulted from direct contiguous spread nor from tumor cell embolization; and (iv) in the presence of generalized lymphatic or hematological malignancy, exclusion of those tumors that metastasized to the lymphatic system [1]. During the decades that followed, some authors reported TTM phenomenon, with the donor neoplasm arising from a variety of sites, including the breast. Although the lung is one of the rarest recipients of TTM [3], renal neoplasms, and particularly ccRCC, are the most common tumor metastasis recipient. Additionally, there have been few cases previously reported of metastatic breast cancer within ccRCC (Table 1).

We reported an extremely rare case of breast cancer metastasis in a lung metastasis of ccRCC that met all Campbell and coworkers' TTM criteria.

Although the exact mechanism by which TTM occurs is yet to be understood, two major theories have been described to explain this phenomenon [12–15]. The first one, the metabolic theory, argues that metastatic tumor cells would preferentially grow in microenvironments with abundant micronutrients, while the second one, the mechanical/anatomic theory, proposes that tumor metastasis is mainly determined by hemodynamic factors of the vascular and lymphatic system, as these factors are most important for successful delivery of metastatic tumor cells. The ccRCC is highly vascularized due to the inactivation of the von Hippel Lindau tumor suppressor gene, which increases hypoxia-inducible factor, leading to increased vascular endothelial growth factor [16]. This, in addition to the fact that kidneys receive approximately 20% of the cardiac output, makes ccRCC a hemodynamically favorable recipient, from a mechanistic perspective [17]. Moreover, and from a metabolic standpoint, ccRCC has increased glycogen and lipid content, which may account for its favorable microenvironment [18]. With this in mind, it is easy to understand why renal cell carcinoma, and in particular ccRCC, is such a frequent recipient of TTM.

Awareness of the TTM phenomenon is important to avoid an incorrect diagnosis and to select the appropriate treatment when unusual malignancies are encountered. In this case, due to their distinct biological behavior, there is not a single effective treatment for both HER2+ breast and ccRC metastatic cancers. Therefore, and since this patient has a good KPS score, both neoplasms were addressed separately. The change of the breast cancer stage led to the alteration of the anti-HER2 treatment strategy, with the initiation of dual blockade, as indicated by Cleopatra trial results for HER2+ metastatic disease [19]. Concerning the ccRCC treatment, our patient was in the International Metastatic Renal Cell Carcinoma Database Consortium (IMRCCDC) favorable risk group. The results of Keynote 426 and of CheckMate 9ER trials support the use of pembrolizumab plus axitinib or nivolumab plus cabozantinib, respectively, as the first-line treatment in metastatic disease for all IMRCCDC risk groups, being the preferred choices in the favorable risk group, but neither of them are reimbursed by the Portuguese Medicines and Heath Care Products Authority (INFARMED), which justify the option of pazopanib administration for this case therapeutic strategy (noninferior to sunitinib in the COMPARZ trial with better quality of life) [20–22].

We report this case for its rarity. To date, literature reported only six cases of metastasis of breast cancer in renal cell carcinomas. Nevertheless, and to the best of our knowledge, this is the first report of a metastasis inside a metastasis.

## Figures and Tables

**Table 1:** Clinical cases of breast carcinoma to renal cell carcinoma tumor-to-tumor metastasis described in the literature.

Gender	Age (years)	Donor histology	Receipt histology	Location	Reference
Female	79	Invasive ductal carcinoma	Clear cell renal cell carcinoma	Right kidney	[6]
Female	62	Invasive ductal carcinoma	Clear cell renal cell carcinoma	Right kidney	[7]
Female	71	Invasive ductal carcinoma	Clear cell renal cell carcinoma	Right kidney	[8]
Female	74	Invasive ductal carcinoma	Chromophobe renal cell carcinoma	Right kidney	[9]
Female	43	Invasive ductal carcinoma	Clear cell renal cell carcinoma	Left kidney	[10]
Female	57	Invasive lobular carcinoma	Clear cell renal cell carcinoma	Right kidney	[11]

## Abbreviations

AC: Doxorubicin and cyclophosphamide
ALD: Axillary lymph node dissection
ccRCC: Clear cell type renal cell carcinoma
CHT: Chemotherapy
CT: Computed tomography
D: Docetaxel
EBRT: External beam radiation therapy
ER: Estrogen receptor
18F-FDG: 18-F-Fluoro-2-deoxyglucose
FISH: Fluorescence in situ hybridization
H&E: Hematoxylin and eosin
HER2: Human epidermal growth factor receptor 2
IDC: Invasive ductal carcinoma
IMRCCDC: International Metastatic Renal Cell Carcinoma Database Consortium
KPS: Karnofsky performance status
MDT: Multidisciplinary team
P: Pertuzumab
PET/CT: Positron emission computed tomography
PgR: Progesterone receptor
T: Trastuzumab
TTM: Tumor-to-tumor metastasis.
